# Temporal Stability of Management Zone Patterns: Case Study with Contact and Non-Contact Soil Electrical Conductivity Sensors in Dryland Pastures

**DOI:** 10.3390/s24051623

**Published:** 2024-03-01

**Authors:** João Serrano, Shakib Shahidian, José Marques da Silva, Luís L. Paniágua, Francisco J. Rebollo, Francisco J. Moral

**Affiliations:** 1 MED—Mediterranean Institute for Agriculture, Environment and Development and CHANGE—Global Change and Sustainability Institute, Universidade de Évora, Pólo da Mitra, Ap. 94, 7006-554 Évora, Portugal; shakib9@gmail.com (S.S.); jmsilva@uevora.pt (J.M.d.S.); 2 Departamento de Ingeniería del Medio Agronómico y Forestal, Escuela de Ingenierías Agrarias, Universidad de Extremadura, Avenida Adolfo Suárez, S/N, 06007 Badajoz, Spain; llpsimon@unex.es; 3 Departamento de Expresión Gráfica, Escuela de Ingenierías Industriales, Universidad de Extremadura, Avenida de Elvas s/n, 06006 Badajoz, Spain; frebollo@unex.es (F.J.R.); fjmoral@unex.es (F.J.M.)

**Keywords:** pastures, soil variability, sensors, electrical conductivity, management zones

## Abstract

Precision agriculture (PA) intends to validate technological tools that capture soil and crop spatial variability, which constitute the basis for the establishment of differentiated management zones (MZs). Soil apparent electrical conductivity (EC_a_) sensors are commonly used to survey soil spatial variability. It is essential for surveys to have temporal stability to ensure correct medium- and long-term decisions. The aim of this study was to assess the temporal stability of MZ patterns using different types of EC_a_ sensors, namely an EC_a_ contact-type sensor (Veris 2000 XA, Veris Technologies, Salina, KS, USA) and an electromagnetic induction sensor (EM-38, Geonics Ltd., Mississauga, ON, Canada). These sensors were used in four fields of dryland pastures in the Alentejo region of Portugal. The first survey was carried out in October 2018, and the second was carried out in September 2020. Data processing involved synchronizing the geographic coordinates obtained using the two types of sensors in each location and establishing MZs based on a geostatistical analysis of elevation and EC_a_ data. Although the basic technologies have different principles (contact versus non-contact sensors), the surveys were carried out at different soil moisture conditions and were temporarily separated (about 2 years); the EC_a_ measurements showed statistically significant correlations in all experimental fields (correlation coefficients between 0.449 and 0.618), which were reflected in the spatially stable patterns of the MZ maps (averaging 52% of the total area across the four experimental fields). These results provide perspectives for future developments, which will need to occur in the creation of algorithms that allow the spatial variability and temporal stability of EC_a_ to be validated through smart soil sampling and analysis to generate recommendations for sustained soil amendment or fertilization.

## 1. Introduction

Several studies show the high spatial variability of soil properties in agricultural fields [1,2]. Previous research has shown that the amount of soil variability across a farm and within a field is of key importance to determine the potential benefits of adopting effective management strategies [3,4]. Understanding this spatial variability is the first step for site-specific crop management [5]. On the other hand, spatial variability and temporal stability are two essential conditions for the adoption of differential management strategies and are the bases for variable rate technology (VRT) implementation [6]. However, relatively little is known about the degree of within-field spatial variation in soils used for livestock production, which leads to the common practice of uniform field management [2]. Site-specific crop management aims to increase profitability and reduce the negative environmental impact of modern farming [7].

Numerous studies use data from sensors to map soil physical and chemical properties to divide the field into smaller, more homogeneous areas (management zones, MZs) [8,9]. Surveying soil spatial variability is the basis for identifying within-field areas of soil similarity, defining MZs, and supporting decision making, for example, to decide on the locations of direct soil sampling (smart soil sampling) [10] or for variable rate application (VRA) of agricultural inputs [1,2,11,12,13]. These subfield regions constitute areas that have similar permanent characteristics, such as topography and nutrient levels [14]. Typically, soil sampling of a field comprises a grid-sampling approach as well as laboratory work [5]. This is impractical at the farming scale because it requires many soil samples in order to achieve a good representation of the soil spatial patterns, and it is labor intensive, time consuming, and expensive [1,3,5]. Therefore, it is desirable to find other more rapid and low-cost means of obtaining information for detailed soil mapping [3].

During previous decades, great progress has been made in the research and development of proximal soil sensing and mapping [9]. The use of geospatial measurements of apparent soil electrical conductivity (EC_a_), combined with global navigation satellite systems (GNSSs) and geographical information systems (GISs), has become instrumental in characterizing the spatial patterns of soil properties within fields [5,14,15]. There are two types of electrical conductivity sensors that are currently on the market: (i) contact sensors, such as the Veris 2000 XA (Veris Technologies, Salina, KS, USA) sensor, which use electrodes, in the shape of coulters, that make contact with the soil to measure the electrical resistivity (the inverse of electrical conductivity); and (ii) non-contact sensors, such as the Dualem 1S (Dualem, Inc., Milton, ON, Canada) or the EM-38 (Geonics Ltd., Mississauga, ON, Canada) sensors, which are based on the principle of electromagnetic induction [14]. Both have advantages and disadvantages, and the different operating principles of these two types of sensors should be considered in the selection of EC_a_ sensing systems for each application [12]. It is generally recognized that both types of sensors represent practical tools to delineate soil-based MZs [11].

Soil electrical conductivity has been frequently used in the establishment of soil MZs and in the inference of several edaphic physicochemical properties and their respective spatial variations [5,16]. Many studies carried out on agricultural soils have reported the relationship of EC_a_ with other soil attributes, including salinity, texture, depth, pH, moisture, organic matter, and cation exchange capacity [5,13]. Changes in the spatial distribution and magnitude of dynamic factors, such as moisture and temperature, can consequently affect the spatial patterns of proximal soil sensing data and their relationship with static soil properties, such as texture [9]. According to Farahani and Buchleiter [17], although the magnitudes of the absolute values of EC_a_ may change in response to modifications in the soil dynamic properties, it is expected that the pattern of EC_a_ spatial variability will not change significantly over time [13]. It is therefore essential that EC_a_ measurements portray the soil’s spatial variability pattern, expressed in terms of delineation of MZ, but to also do so in a stable way over time [13], guaranteeing the sustainability of management decisions in the medium and long term [2]. Considering that an MZ is often used for several years, the variables should be temporally stable [18].

Several previous studies have evaluated and reported the temporal stability of EC_a_. Some have reported weak temporal associations [11], and others have shown that EC_a_ has temporal stability [2,19,20]; however, there are no known studies evaluating the temporal stability of MZ, especially when obtained from measurements using sensors with different principles.

The aim of this study was to assess the temporal stability of MZ patterns using two different types of EC_a_ sensors, namely a contact-type (Veris 2000 XA) and an electromagnetic induction type (EM-38), after approximately 2 years in four fields of dryland pastures in the Alentejo region of Portugal.

## 2. Materials and Methods

### 2.1. Description of Experimental Fields

The experimental work was carried out at four fields, namely two in the “Mitra” experimental farm (“ECO” and “MIT”), one in the “Murteiras” farm (“MUR”), and another in the “Padres” farm (“PAD”), which are all permanent and biodiverse dryland pastures of the Montado ecosystem, located in Alentejo, in the district of Évora, in southern Portugal (Table 1).

In this mixed ecosystem, the predominant trees are Holm oak trees, and the main animal species are cows and sheep in extensive grazing. The soil type is Cambisol, originating from granite [21], and is characterized by a coarse texture. These soils are not highly fertile and are primarily utilized for mixed agro-silvo-pastoral systems [2]. The location of these fields is representative of the temperate climatic conditions of Portugal (classified as ‘Csa’: hot summer Mediterranean climate according to the Köppen–Geiger climate classification). The temperature ranges between 0 °C as the minimum in winter and 40 °C as the maximum in summer. The mean annual rainfall is 567 mm, with precipitation mainly concentrated between October and April, and practically non-existent during the summer (source: Portuguese Institute of Sea and Atmosphere) [22].

### 2.2. Topographic and Soil Electrical Conductivity Surveys

**Figure 1:**
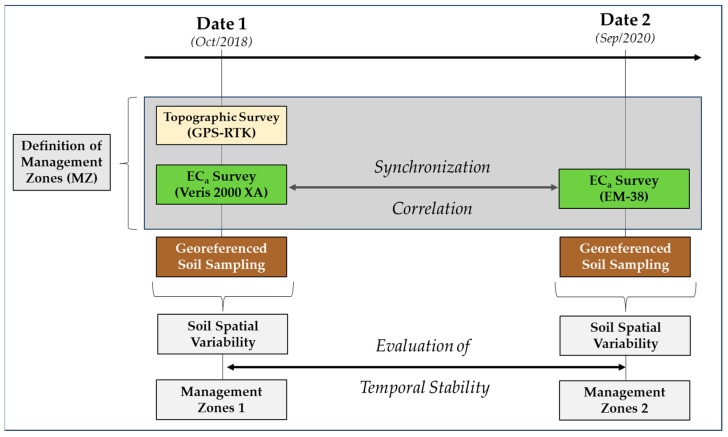
Schematic representation of the experimental approach proposed in this study.

The experimental approach proposed in this study is shown in Figure 1.

**Figure 2:**
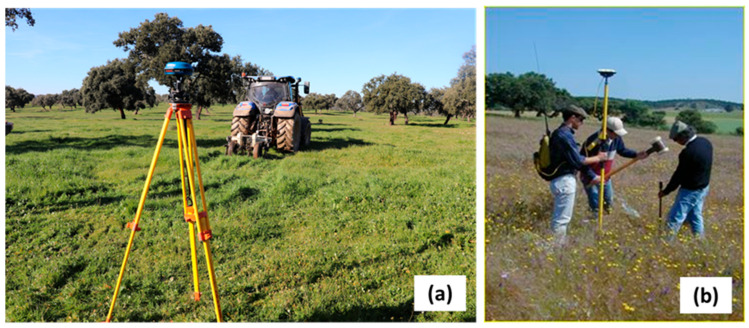
Trimble RTK/PP-4700 GNSS: (**a**) differential correction station and tractor-mounted rover; (**b**) rover to georeference soil sampling points.

A topographic survey of the four experimental fields was carried out using a Real-Time Kinematic (RTK) GNSS instrument (Trimble RTK/PP-4700 GNSS, Trimble Navigation Limited, Sunnyvale, CA, USA). The elevation data were sampled in the field with the GNSS antenna assembled on a tractor (Figure 2). For each field, the digital elevation model (DEM) was generated using the triangulated irregular network (TIN) interpolation tool from ArcGIS 9.3. The TIN algorithm uses sample points to create a surface formed by triangles based on nearest neighbor point information. This vector information was converted into a grid surface with a 1 m resolution using the “Spatial Analyst” tool.

**Figure 3:**
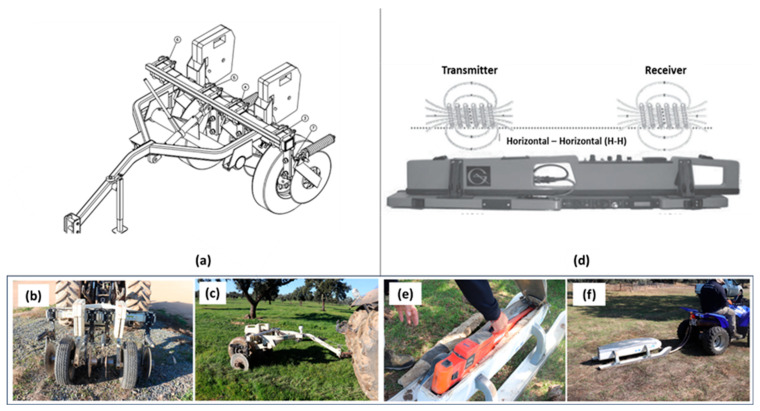
Veris 2000 XA contact-type sensor (**a**–**c**) and EM38 non-contact sensor (**d**–**f**).

The EC_a_ in the four fields was measured using a Veris 2000 XA contact-type sensor (Veris Technologies, Salina, KS, USA) in October 2018 (Figure 3a–c). This sensor was mounted on a chassis supported by two wheels, and its active components consisted of two pairs of coulter-electrodes—adjustable rotating discs. One pair injected a current into the soil (outermost discs), while the other pair (innermost discs) measured the voltage drop.

The adjustment of the discs generates a set of topsoil data (from 0 to 0.30 m in depth). The sensor, equipped with a GNSS antenna, was towed by a tractor at an average speed of 2.0 m s^−1^, and successive passes spaced 10 m apart were made across the field. The EC_a_ measurements were recorded every second, resulting in a spatial resolution of a 2 m by 10 m grid.

The EC_a_ was also measured using an EM38 non-contact sensor (Geonics Ltd., Mississauga, ON, Canada). It was employed in the horizontal dipole orientation to perform EC_a_ surveys in each pasture field in September 2020 (Figure 3d–f). The two receiver coils were positioned 0.5 m away from the transmitter. Although this sensor provided data from depth ranges of 0.75 m and 0.375 m, only the topsoil data in the range of 0–0.375 m were utilized in this study. The device was towed by an all-terrain vehicle equipped with a GNSS antenna. The vehicle maintained an average speed of 2.5 m s^−1^, making successive passes across the fields and recording the EC_a_ measurements every second. From these EC_a_ data, Kriged EC_a_ maps were generated for each pasture field using the collected EC_a_ data. The EC_a_ values at each sample location were then extracted from these EC_a_ maps.

The estimation of EC_a_ at unsampled locations was performed using the ordinary point kriging algorithm, which integrates the spatial correlation structure described with the variograms. The resulting kriged maps show the spatial distribution of EC_a_ in the experimental field. Finally, the kriged EC_a_ maps were generated using the ArcMap module of ArcGIS.

### 2.3. Soil Sampling and Analysis

**Figure 4:**
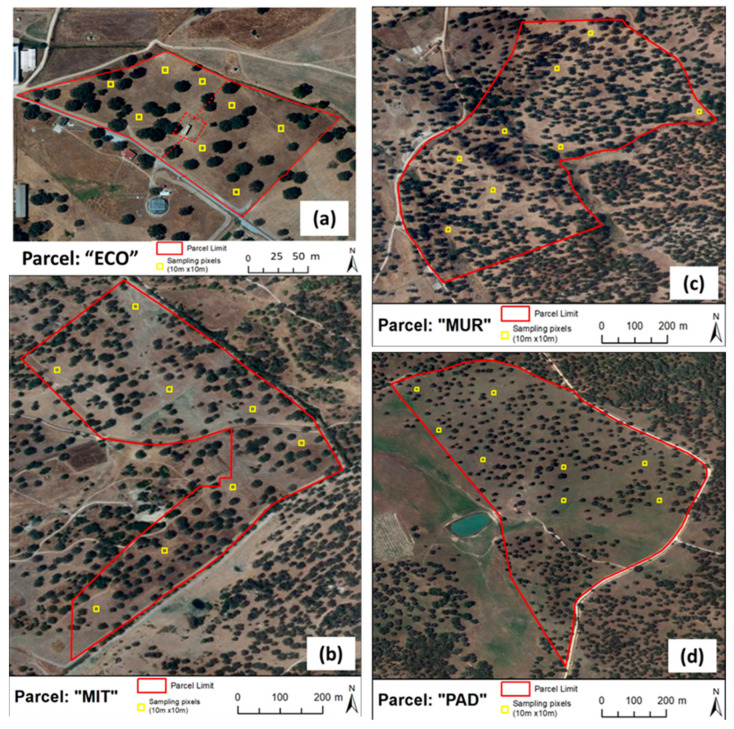
Eight georeferenced soil samples in each experimental field: “ECO” (**a**), “MIT” (**b**), “MUR” (**c**), and “PAD” (**d**).

On both dates and in each experimental field, following the EC_a_ measurements, eight georeferenced composite soil samples (Figure 4) were collected at depths ranging from 0 to 0.30 m using a gouge auger and a hammer. Each composite sample resulted from the combination of five sub-samples collected within an approximately 10 m × 10 m area: one from the center of the sampling area and the other four from the respective quadrant. Soil samples were transported to the laboratory in metallic boxes, weighed, and then dried at 105 °C until a constant weight was achieved. Once cooled, they were weighed to establish the mean soil moisture content (SMC). Subsequently, the fine components of the soil (fraction with a diameter < 2 mm) underwent chemical analyses using standard reference laboratory methods [23]. The pH (in a 1:2.5 soil-to-water suspension) was determined using the potentiometric method. Organic matter (OM) was measured by combustion and CO_2_ measurement, employing an infrared detection cell. Phosphorous (P_2_O_5_) and potassium (K_2_O) were extracted using the Egner–Riehm method, with P_2_O_5_ measured using the colorimetric method and K_2_O measured with a flame photometer.

On date 2 (September 2020), soil samples were also processed for particle size distribution using a sedimentographer (Sedigraph 5100, manufactured by Micromeritics). Additionally, samples were processed for determination of cation exchange capacity (CEC), extracted with ammonium acetate.

### 2.4. Statistical Analysis and Data Processing

With the aim of assessing the soil spatial variability, an initial descriptive statistical analysis (mean, standard deviation, and range) was carried out for the measured parameters.

**Figure 5:**
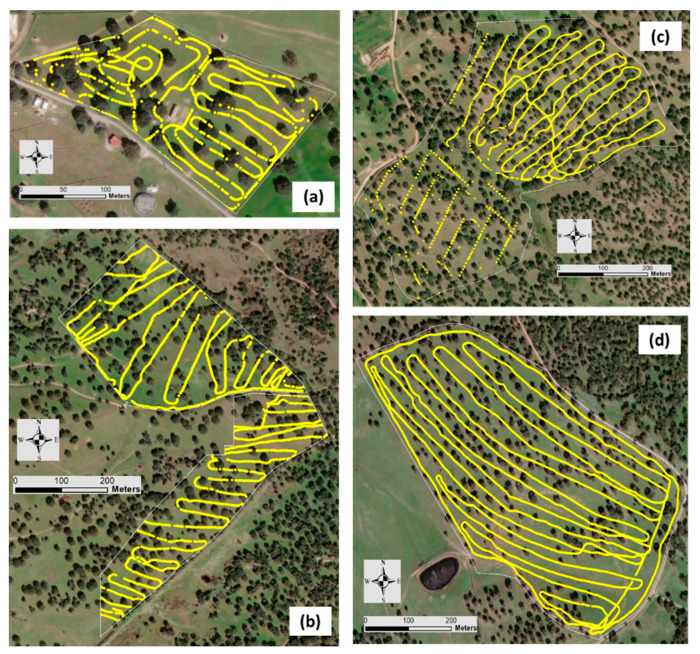
Paths taken by Veris 2000 XA sensor in each experimental field: “ECO” (**a**), “MIT” (**b**), “MUR” (**c**) and “PAD” (**d**).

**Figure 6:**
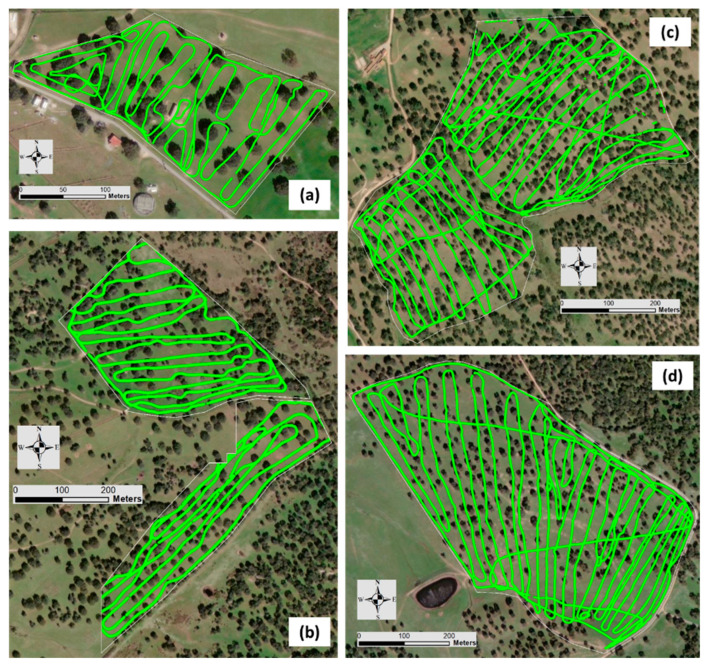
Paths taken by EM-38 sensor in each experimental field: “ECO” (**a**), “MIT” (**b**), “MUR” (**c**), and “PAD” (**d**).

The data of EC_a_ obtained by a Veris sensor in October 2018 and by EM-38 in September 2020 were synchronized using the geographic coordinates of each point to evaluate the temporal stability of the EC_a_ measurements. Figure 5 and Figure 6 show the paths taken by the two sensors in each experimental field, Veris 2000 XA and EM-38, respectively.

A combined dataset was created for each date: each Veris data point obtained was paired with the nearest data point obtained by EM-38 based on GNSS coordinates. If a match in spatial coordinates was not found within a 5 m radius, that point was removed from the dataset—a procedure similar to that used by Sudduth et al. [15]. The sets of data, that is, points with common geographic coordinates in both surveys (date 1 and date 2) in each experimental field underwent linear correlation analysis, which was conducted using the IBM SPSS statistical package (version 25, IBM Corp, Armonk, NY, USA), to obtain the Pearson correlation coefficients (r) using the method of least squares (*p* < 0.05).

The EC_a_ results obtained on each date, combined with elevation data (survey conducted on the first date), served as the input for the geostatistical analyses, enabling the delineation of management zones (MZ) for each date and experimental field. These homogeneous subfields were determined using a fuzzy cluster algorithm [24], and the MZ Analyst (MZA) software (Microsoft Corp., Redmond, WA, USA) was employed in this study. The procedures for delineating and evaluating the number of MZs used in this software were described by Fridgen et al. [25]. From a practical standpoint, three MZs with different productive potential (less, intermediate, and more potential) were considered in each experimental field.

In order to analyze the temporal stability of the MZ defined in each field, a comparison was carried out between each pixel of the MZ maps based on the EC_a_ measurements made with both sensors. Thus, for each field, a reclassification process was performed on the two MZ maps, assigning the value 0 to the pixels of the MZ with higher potential, 1 to those with intermediate potential, and 2 to those with low potential. Subsequently, using map algebra, both MZ maps were overlaid, producing a new map distinguishing three possible zones: one where pixels have a value of 0, meaning that the MZ coincides on both maps; another where pixels have a value of 1, meaning that there is a difference of 1 between the values of the pixel on the two maps; and a third zone with pixels whose value is 2, meaning that the pixel of the two maps have a difference of 2 between them. In this new map, the zone containing pixels with a value of 0 is denominated “stable”, the zone with pixels having a value of 1 is called “unstable”, and the zone containing pixels with a value of 2 is called “very unstable”.

## 3. Results and Discussion

### 3.1. Soil Characteristics: Spatial and Temporal Variability

The soil moisture (Table 2) was low in both surveys carried out at the ECO and MIT fields (between 5.5 and 8.1%) with a coefficient of variation (CV) below 30%. In the case of the MUR and PAD fields, the first surveys (date 1) were carried out with much higher soil moisture values (17% in MUR and 19% in PAD) than those in the second surveys (10% in MUR and 7% in PAD).

The EC_a_ values were significantly higher in the measurements carried out in all plots on date 2 (Table 2) with the EM-38 sensor when compared to the measurements carried out on date 1 with the Veris 2000 XA sensor. The measurements taken with Veris depend on the adequate penetration of the discs (and their respective electrodes), presenting greater difficulty in soils with reduced soil moisture, relatively compacted soils (subject to animal trampling), and soils with stones (as is the case). Measurements with non-contact induction sensors overcome these difficulties and therefore seem better adapted to measurements in dryland pastures. The measurements with Veris showed a CV of approximately 50–70%, while the measurements with EM-38 showed a systematically lower CV, which ranged between 15 and 50%.

The results obtained in this study (Table 3) confirm this variability, reflected in the CV and intra and inter experimental fields, and highlight some of the main limitations of these soils; in general, these include a low pH (slightly acidic with low CV, <10%), usually with a coarse texture, a relatively low OM content (between 1.3 and 2.7%, with a CV between 7 and 35%), low amounts of phosphorous (P_2_O_5_ < 30 mg kg^−1^), and medium or high levels of potassium (generally greater than 45 mg kg^−1^ and, in some cases, greater than 100 mg kg^−1^, with a relatively high CV, generally >30%), which are aspects that were already mentioned in another study [22].

**Figure 7:**
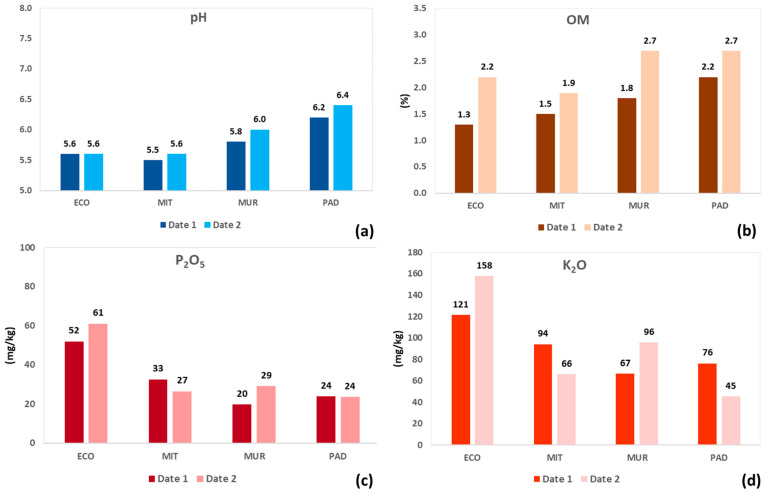
Temporal evolution of soil parameters in each experimental field between sampling dates (date 1 versus date 2): pH (**a**); organic matter, OM (**b**); phosphorous, P_2_O_5_ (**c**); and potassium, K_2_O (**d**).

A comparison between soil sampling dates (Figure 7) highlights the trend for higher OM contents on date 2 relative to date 1 in all four fields. In regard to the soil parameters (Table 4), the low clay contents are evident (between 4 and 8.5% in the four fields together), revealing a coarse texture (between loamy sand and sandy loam) with a relatively high CV (between 17 and 55%). The CEC is relatively low (on average, between 7 and 9 cmol kg^−1^), except in the field of PAD, where the average value is approximately double, with the CV being very variable (approximately between 10 and 60%).

The spatial and temporal variability normally associated with soil parameters is in line with what has been reported in other research studies and is particularly accentuated in the Montado by the presence of trees and grazing animals [6,26]. The combined effects of an undulated landscape, with sparse trees and animals that selectively graze the plant species and make a heterogeneous deposition of dung, cause notable spatial and temporal variability in the soil nutrient concentration [26]. A high CV of soil properties normally indicates high spatial variability and consequently suggests the convenience of site-specific management [14].

On the other hand, soil spatial variability and temporal stability are two essential conditions for the definition of an MZ [9] and for adopting precision agriculture (PA) strategies [1,2,11,12,13].

### 3.2. Correlation between Soil Apparent Electrical Conductivity (EC_a_) Measurements

**Figure 8:**
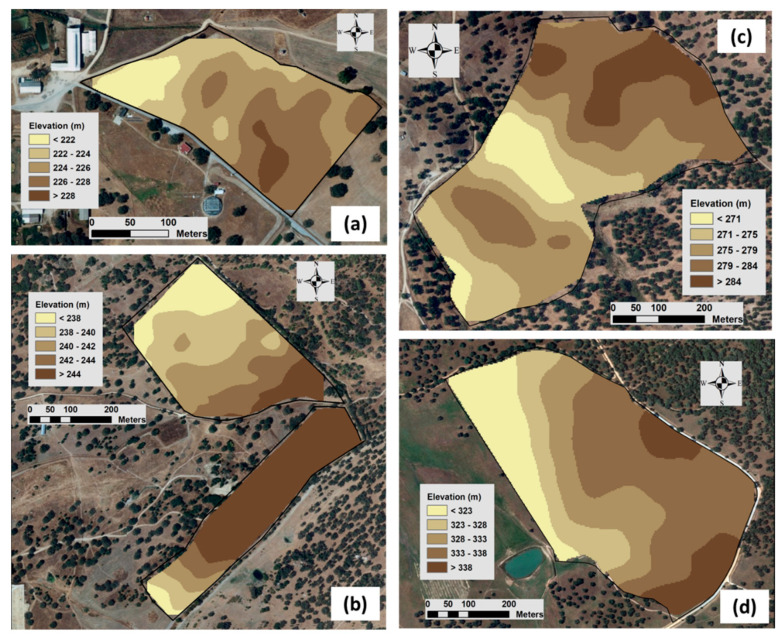
Elevation maps of each experimental field: “ECO” (**a**), “MIT” (**b**), “MUR” (**c**), and “PAD” (**d**).

Figure 8 shows the maps of spatial variability of elevation in each experimental field. The wavy relief characteristic of this region [27], with higher areas of reduced amplitude alternating with small valleys, is presented.

**Figure 9:**
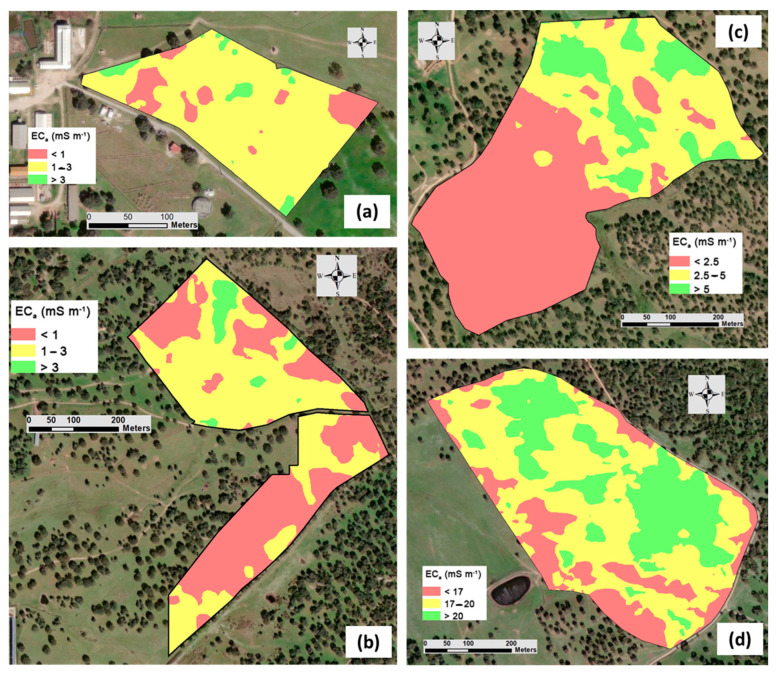
Apparent soil electrical conductivity maps obtained using Veris 2000 XA sensor in each experimental field: “ECO” (**a**), “MIT” (**b**), “MUR” (**c**), and “PAD” (**d**).

**Figure 10:**
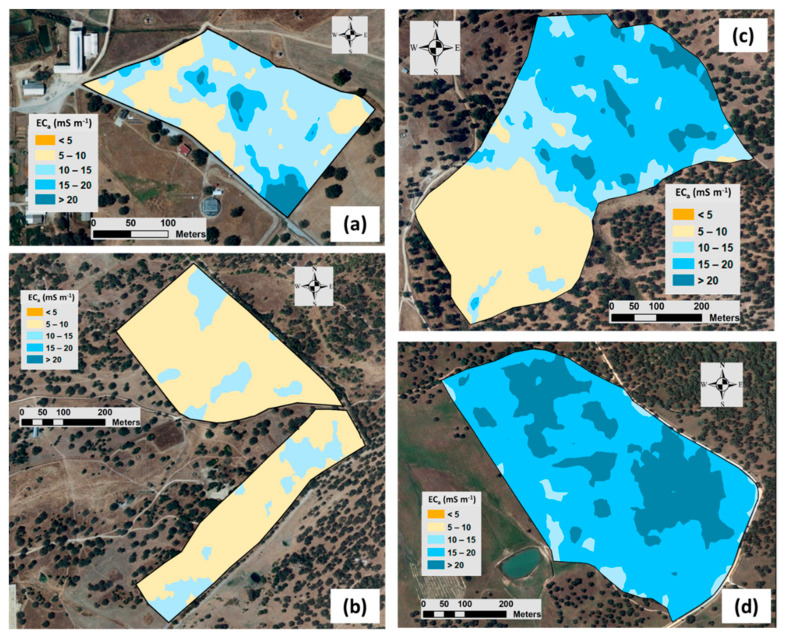
Apparent soil electrical conductivity maps obtained using EM-38 sensor in each experimental field: “ECO” (**a**), “MIT” (**b**), “MUR” (**c**), and “PAD” (**d**).

Figure 9 and Figure 10 show, respectively, the spatial variability of EC_a_ obtained from the survey carried out with the Veris sensor and with the EM-38 sensor in each experimental field. The maps of EC_a_ of date 1 show a small amplitude in the ECO and MIT fields, reflecting the low SMC and its implication on the operation of the contact sensor (Veris 2000 XA), unlike what occurred in the MUR and PAD fields, where the SMC was relatively high. The maps of EC_a_ of date 2 show, in general, greater amplitude and variability, despite the more homogeneous SMC across the four experimental fields. In general, visual assessments of the EC_a_ spatiotemporal variability maps revealed a similar pattern of spatial variability on different dates in all fields, which was also evidenced by Medeiros et al. [13].

**Figure 11:**
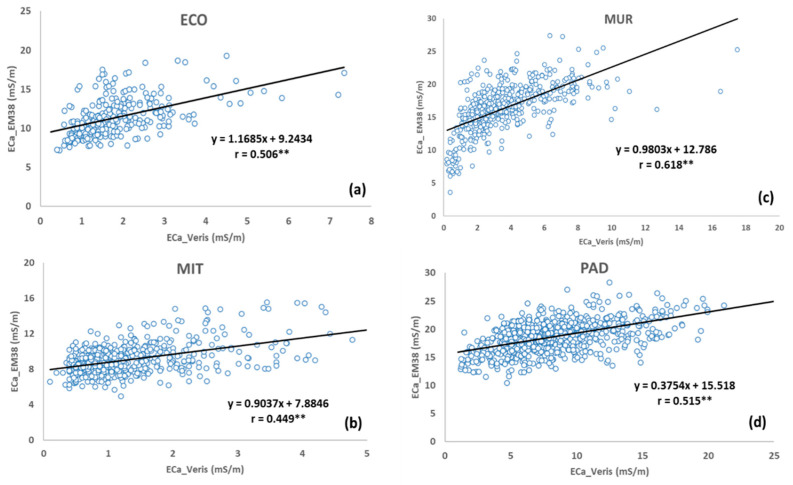
Correlation between soil apparent electrical conductivity (EC_a_) measured using Veris 2000 XA and EM-38 sensors in each experimental field: “ECO” (**a**), “MIT” (**b**), “MUR” (**c**), and “PAD” (**d**). ** Statistically significant at 99% confidence level (*p* < 0.01).

The correlation between the EC_a_ measurements in each date, after coordinate synchronization, for each experimental field is shown in Figure 11: ECO (a), MIT (b), MUR (c), and PAD (d). All correlations were statistically significant (*p* < 0.01), with very interesting correlation coefficients, between 0.449 (MIT field) and 0.618 (MUR field).

The spatial regressions and paired t-tests applied by McCutcheon et al. [11] to investigate differences between the EC_a_ values from different measurement dates (temporal persistence) with a contact sensor (Veris) provided correlation coefficients with a white range of variation (between 0.10 and 0.76). Martini et al. [20] compared repeated EMI measurements with high-resolution soil moisture and temperature data and reported that the spatial patterns of EC_a_ differed from those of the soil water content and that the relation between both variables was changing over time. Various correction or standardization methods have been proposed, and it has been recommended that measurements be made under similar conditions; however, this is often not feasible in practice in fields where conditions change on small spatial scales [9]. By using an EMI device four times (Dualem 1S), Serrano et al. [2] found temporally stable spatial EC_a_ patterns in a Mediterranean pasture over a period of 7 years despite changing environmental and management conditions. Various studies have demonstrated the possibilities for significant changes in the measured EC_a_ over time, nevertheless, with relatively stable spatial structure representations [7].

In this study, the smallest number of points obtained by coordinate synchronization was found in the ECO field, which is a small field compared to the others (only 4 ha, while the rest have areas of 20 to 30 ha). The MUR field also has a smaller number of points, since in the southwestern area, the Veris sensor faced some measurement problems (Figure 5c), which can be associated with the presence of stones on the surface, which interrupt the contact between the electrodes and the ground and reinforces the aforementioned difficulty of contact sensors in stony soils [12]. It is important to note that the surveys were carried out by independent service providers. The results would have certainly improved if the sensor transects in the second survey had followed the paths of the first survey in each field, which would have allowed a much greater number of points as a result of synchronization and thus would have strengthened the correlation.

### 3.3. Temporal Stability of Management Zones

**Figure 12:**
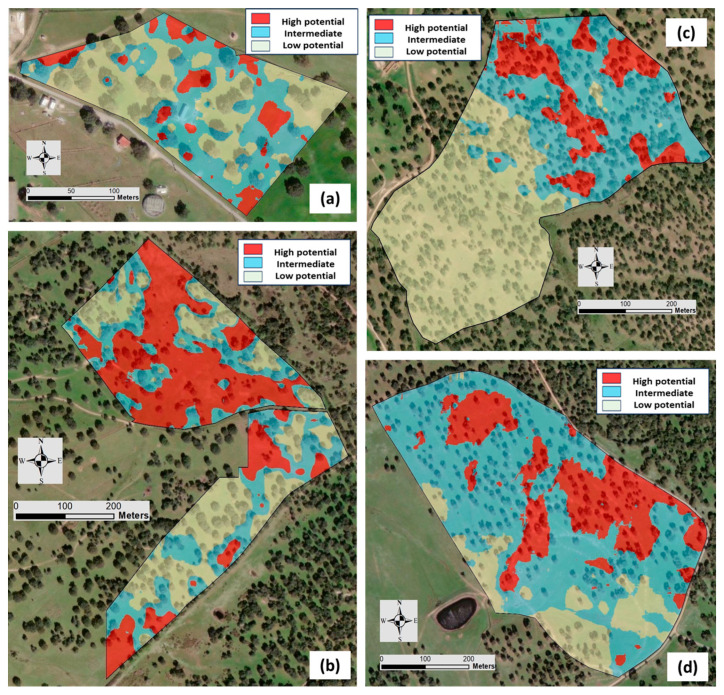
Management zone (MZ) maps of each experimental field based on the elevation and Veris 2000 XA sensor measurements: “ECO” (**a**), “MIT” (**b**), “MUR” (**c**), and “PAD” (**d**).

**Figure 13:**
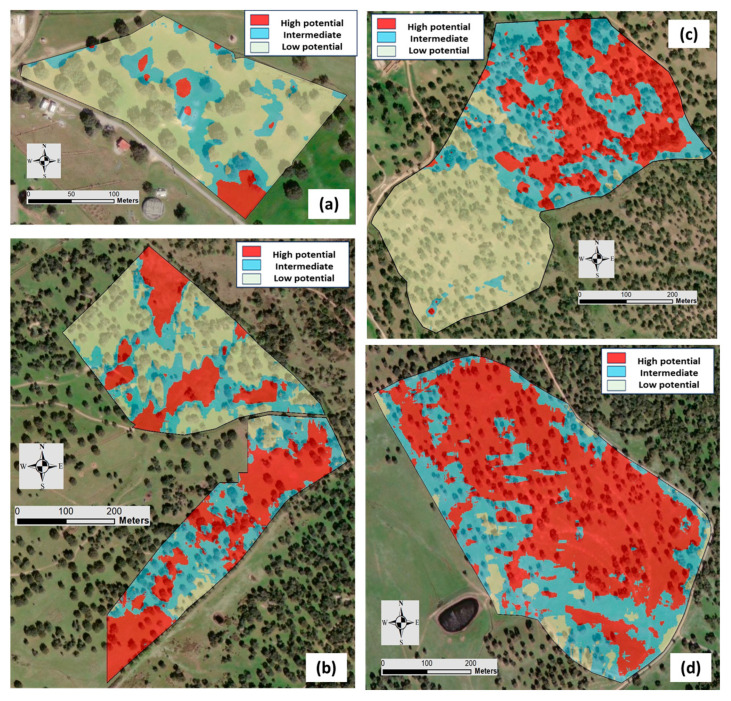
Management zone (MZ) maps of each experimental field based on the elevation and EM-38 sensor measurements: “ECO” (**a**), “MIT” (**b**), “MUR” (**c**), and “PAD” (**d**).

The application of geostatistical algorithms based on elevation and EC_a_ data allowed for the MZs and the correspondent maps in each date to de defined (Figure 12 and Figure 13, respectively) and in each experimental field: ECO (a), MIT (b), MUR (c), and PAD (d).

In general, the spatial patterns of MZ maps reflect the gradients of EC_a_ in each field and are similar across measurement dates. Table 5 shows the area of each MZ (low, intermediate, and high potential) in each date, in percentage of the total area of each field.

**Figure 14:**
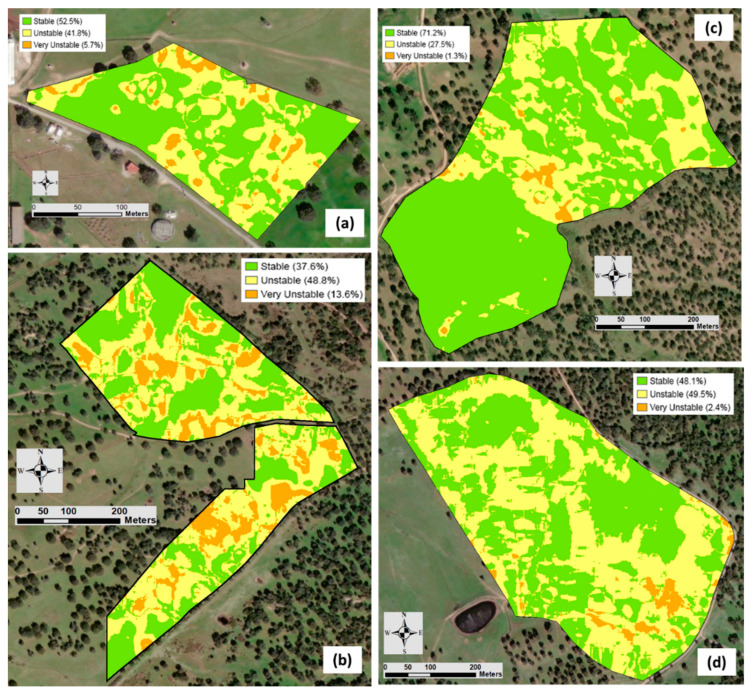
Maps of temporal stability of management zones (MZ) of each experimental field: “ECO” (**a**), “MIT” (**b**), “MUR” (**c**), and “PAD” (**d**). Stable, unstable, and very unstable areas are indicated as percentages of total area.

A number of methods have been used by different research teams to define the spatial and temporal trends found within a field [28,29,30]. In this study, the temporal stability of an MZ (Table 6) was evaluated by an overlap reclassification process of MZ maps, allowing for the definition of three new zones, namely stable, unstable, and very unstable, expressed as percentages of the total area of each field. In average, 52% of the area presented temporal stability (in the same MZ category in both EC_a_ surveys, with a minimum value of 37.6% in the MIT field and a maximum of 71.2% in the MUR field), 42% of the area presented temporal instability (in the next or previous category in both EC_a_ surveys), and only 6% presented very temporal instability (two categories above or below in both EC_a_ surveys). This information, presented spatially through temporal stability maps (Figure 14), provides good prospects for the use of EC_a_ in the medium- and long-term definition of MZ. If a spatial pattern is temporally stable within a field, then it is reasonable to suppose that it should be a reasonably good predictor of the spatial patterns in the following years. This assumes that the prevailing conditions and limiting factors are consistent over the years [28].

The results of this study, especially those relating to the temporal stability of the MZ patterns obtained by different sensors, are very encouraging. However, various authors have shown inconsistent relationships between the EC_a_ and soil characteristics, probably due to the fact that the EC_a_ is influenced by various complex interactions between site-dependent soil properties [1,14,16,31].

Despite the temporal stability of the EC_a_ patterns, in relative terms, reflected in the temporal stability of the patterns of the MZ maps, it will be interesting, in future works, to extend the findings to other types of soils (in particular, with finer textures) and conditions (namely different SMCs) to identify the source of the discrepancy in absolute terms in the measurement of the EC_a_ using different sensors (contact versus non-contact), with systematically higher values obtained using the EMI sensor (EM-38 in this case) compared to the contact sensor (Veris 2000 XA in this case).

These results also suggest a next step in the logic of validating the MZ obtained by each of the sensors, either through soil smart sampling carried out in each MZ [14] or through the evaluation of vegetation indices, or VIs (namely the NDVI), which is an approach that has been followed in several research works [32]. Through a soil analysis, it will be possible to find the relationship between EC_a_ measurements and soil parameters with potential for site-specific management in dryland pastures, for example, in terms of the differentiated application of lime or phosphorus amendments. To transpose these MZ maps to amendment or fertilization prescription maps, it is fundamental to develop algorithms to evaluate the agronomic significance of this classification (MZ) and establish more general methods of mapping and quantifying variable input prescriptions, for example, lime amendment and nitrogen or phosphorous fertilizer application [33]. On the other hand, there is a growing interest in rapid MZ validation methodologies, such as those based on VIs obtained from remote sensing, by recovering the time-series of the NDVI (for example) throughout the vegetative cycle, which is related with pasture productivity and quality, and complementing and improving the rigor of the validation obtained from smart soil sampling [32].

Given that this is an exploratory study that was carried out in a relatively restricted soil type under specific coarse-textured soils, further studies using different soil types should be conducted.

## 4. Conclusions

The results obtained in this study show an important spatial variability in the soil parameters in all experimental fields. In temporal terms, the tendency for there to be an increase in the organic matter levels between sampling dates is noteworthy. In regard to the soil apparent electrical conductivity parameter, the significant correlation between measurements (different dates and sensors) after coordinate synchronization in all experimental fields (with correlation coefficients between 0.449 and 0.618) is of great importance.

Based on a geostatistical analysis of the soil electrical conductivity and topographic surveys, three management zones were defined (with high, intermediate, and low potential). The soil electrical conductivity measurements with different sensors (contact and non-contact), carried out almost two years apart, revealed spatial patterns in management zones with remarkable temporal stability (averaging 52% of the total area across four fields), which is a good indicator of potential for the use of soil electrical conductivity in medium- and long-term management decisions.

These results suggest that future developments should focus on creating algorithms to validate the spatial variability and temporal stability of soil electrical conductivity through smart soil sampling, extending the database and thereby enhancing the process of recommendations for sustained soil amendment or fertilization.

## Figures and Tables

**Table 1:** The main characteristics of the four experimental fields used in this work.

Field Code	Coordinates	Area (ha)	Soil Texture	Animal Species (Type of Grazing)
ECO	38°53.10′ N; 8°01.10′ W	4.3	Loamy sand	Sheep (Rotational grazing)
MIT	38°32.17′ N; 7°59.83′ W	20.2	Loamy sand	Cattle (Rotational grazing)
MUR	38°23.4′ N; 7°52.5′ W	29.6	Sandy loam	Sheep (Permanent grazing)
PAD	38°36.4′ N; 8°8.7′ W	32.2	Sandy loam	Cattle (Permanent grazing)

**Table 2:** Descriptive statistics (mean ± standard deviation and range) of the soil apparent electrical conductivity (EC_a_) and soil moisture content (SMC) in each of the four experimental fields used in this work on both dates.

Field	ECO	ECO	MIT	MIT	MUR	MUR	PAD	PAD
Date	1	2	1	2	1	2	1	2
EC_a_ (mS m^−1^)								
Mean ± SD	2.1 ± 1.2	12.2 ± 4.6	1.4 ± 1.0	9.1 ± 4.6	3.9 ± 2.4	13.8 ± 5.4	8.5 ± 4.1	18.6 ± 2.9
Range	0.2–12.1	4.7–36.5	0.1–9.6	1.3–130.0	0.2–21.2	0.9–33.9	0.7–54.0	0.1–32.5
SMC (%)								
Mean ± SD	6.9 ± 2.0	5.5 ± 0.7	8.1 ± 1.0	7.6 ± 1.6	16.8 ± 5.1	10.0 ± 2.3	18.8 ± 2.4	6.6 ± 1.4
Range	4.4–10.2	4.7–6.8	6.4–9.8	4.9–9.4	9.7–25.6	6.5–12.9	14.8–22.9	4.3–8.6

**Table 3:** Descriptive statistics (mean ± standard deviation and range) of the soil parameters in each of the four experimental fields used in this work on both dates.

Field	ECO	ECO	MIT	MIT	MUR	MUR	PAD	PAD
Date	1	2	1	2	1	2	1	2
pH								
Mean ± SD	5.6 ± 0.2	5.6 ± 0.3	5.5 ± 0.2	5.6 ± 0.3	5.8 ± 0.5	6.0 ± 0.5	5.8 ± 0.5	6.4 ± 0.5
Range	5.2–5.8	5.2–6.0	5.0–5.8	5.3–6.0	5.0–6.4	5.3–6.6	5.0–6.4	5.7–7.0
OM (%)								
Mean ± SD	1.3 ± 0.2	2.2 ± 0.8	1.5 ± 0.3	1.9 ± 0.4	1.8 ± 0.6	2.7 ± 0.5	1.8 ± 0.6	2.7 ± 0.2
Range	1.0–1.8	1.4–3.1	0.9–2.1	1.3–2.5	1.0–3.2	2.1–3.3	1.0–3.2	2.3–2.8
P_2_O_5_ (mg kg^−1^)								
Mean ± SD	51.9 ± 19.7	61.0 ± 23.1	32.6 ± 21.5	26.5 ± 13.0	19.8 ± 17.9	29.2 ± 21.7	19.8 ± 17.9	23.7 ± 6.7
Range	17.0–88.0	41.0–105.0	7.8–81.0	15.0–45.0	4.0–55.0	10.0–67.0	4.0–55.0	18.0–33.0
K_2_O (mg kg^−1^)								
Mean ± SD	121.3 ± 28.5	158.0 ± 66.7	94.0 ± 72.1	66.3 ± 41.9	66.8 ± 26.1	95.7 ± 27.2	66.8 ± 26.1	45.3 ± 4.1
Range	78.0–184.0	72.0–268.0	18.0–380.0	26.0–142.0	30.0–110.0	58.0–130.0	30.0–110.0	40.0–52.0

OM—organic matter; P_2_O_5_—phosphorus; K_2_O—potassium.

**Table 4:** Descriptive statistics (mean ± standard deviation and range) of the soil texture and cation exchange capacity (CEC) in each experimental field used in this work in date 2.

Parameter	Clay (%)	Silt (%)	Sand (%)	CEC (cmol kg^−1^)
ECO				
Mean ± SD	4.0 ± 0.7	8.0 ± 2.2	88.0 ± 2.8	7.4 ± 1.5
Range	2.7–4.7	3.8–9.8	85.8–93.5	5.7–9.2
MIT				
Mean ± SD	6.8 ± 2.4	9.5 ± 2.5	83.7 ± 3.7	9.4 ± 5.8
Range	3.2–9.2	6.2–13.2	77.6–88.4	5.8–21.2
MUR				
Mean ± SD	8.5 ± 4.7	15.9 ± 10.6	75.6 ± 14.6	8.7 ± 2.8
Range	3.2–17.0	5.1–34.5	48.5–88.3	5.2–12.4
PAD				
Mean ± SD	6.6 ± 2.0	15.4 ± 2.2	78.0 ± 2.6	15.5 ± 1.3
Range	4.6–10.4	13.2–19.2	73.9–80.3	14.3–17.6

**Table 5:** Area of each management zone (MZ) in each date, shown in percentage of the total area of each field.

Field	ECO	ECO	MIT	MIT	MUR	MUR	PAD	PAD
Date	1	2	1	2	1	2	1	2
MZ (%)								
Low Potential	45.7	76.9	28.7	28.5	49.9	27.4	14.3	0.9
Intermediate Potential	41.5	17.2	31.6	37.2	34.9	32.3	60.1	42.3
High Potential	12.8	5.9	39.7	34.3	15.2	40.3	25.6	56.8

**Table 6:** Temporal stability of management zones (MZ) between dates of evaluation, in percentage of total area of each field.

Field	ECO	MIT	MUR	PAD
Temporal stability of MZ (% of area)				
Stable	52.5	37.6	71.2	48.1
Unstable	41.8	48.8	27.5	49.5
Very unstable	5.7	13.6	1.3	2.4

## Data Availability

The data are unavailable due to privacy restrictions.
